# Fluorescence microscopy datasets for training deep neural networks

**DOI:** 10.1093/gigascience/giab032

**Published:** 2021-05-05

**Authors:** Guy M Hagen, Justin Bendesky, Rosa Machado, Tram-Anh Nguyen, Tanmay Kumar, Jonathan Ventura

**Affiliations:** UCCS BioFrontiers Center, University of Colorado at Colorado Springs, 1420 Austin Bluffs Parkway, Colorado Springs, CO 80918, USA; UCCS BioFrontiers Center, University of Colorado at Colorado Springs, 1420 Austin Bluffs Parkway, Colorado Springs, CO 80918, USA; UCCS BioFrontiers Center, University of Colorado at Colorado Springs, 1420 Austin Bluffs Parkway, Colorado Springs, CO 80918, USA; George Mason University, 4400 University Drive, Fairfax, VA 22030, USA; Department of Computer Science and Software Engineering, California Polytechnic State University, San Luis Obispo, CA 93407, USA; Department of Computer Science and Software Engineering, California Polytechnic State University, San Luis Obispo, CA 93407, USA

**Keywords:** fluorescence microscopy, deep learning, convolutional neural networks

## Abstract

**Background:**

Fluorescence microscopy is an important technique in many areas of biological research. Two factors that limit the usefulness and performance of fluorescence microscopy are photobleaching of fluorescent probes during imaging and, when imaging live cells, phototoxicity caused by light exposure. Recently developed methods in machine learning are able to greatly improve the signal-to-noise ratio of acquired images. This allows researchers to record images with much shorter exposure times, which in turn minimizes photobleaching and phototoxicity by reducing the dose of light reaching the sample.

**Findings:**

To use deep learning methods, a large amount of data is needed to train the underlying convolutional neural network. One way to do this involves use of pairs of fluorescence microscopy images acquired with long and short exposure times. We provide high-quality datasets that can be used to train and evaluate deep learning methods under development.

**Conclusion:**

The availability of high-quality data is vital for training convolutional neural networks that are used in current machine learning approaches.

## Data description

### Context

Fluorescence microscopy is an important technique in many areas of biomedical research, but its use can be limited by photobleaching of fluorescent probe molecules caused by the excitation light that is used. In addition, reactive oxygen species that are generated by exposing samples to light can cause cell damage and even cell death, limiting imaging of live cells [1,2]. Many strategies have been devised to overcome this problem including the use of specialized culture media [3,4], pulsed excitation [5], or more elaborate methods such as controlled light exposure microscopy [6,7].

Another approach involves recording of fluorescence microscopy images with short exposure times, low-excitation light intensity, or both. This results in images with low signal-to-noise ratios (SNRs), which can then be improved using a variety of image restoration approaches [8–12]. Noise in low-light images of this type typically follows a Poisson-Gaussian distribution. This condition makes solving the inverse problem that arises in image restoration methods difficult and has led to a number of approximate methods [13].

Recently, deep learning methods in artificial intelligence [14] have been applied to many problems in image analysis, including those in optical microscopy [15–18] and in image denoising [19–21]. Deep learning approaches typically require a large amount of data to train the underlying convolutional neural network [22]; however, such datasets are not always available. Here we provide fluorescence microscopy datasets that can be used to train and evaluate neural networks for the purpose of image denoising. The dataset consists of pairs of images acquired with different exposure times (or in the case of confocal microscopy, different laser power and detector gain settings). After training, the network can subsequently be used to enhance the SNR of newly acquired images.

One advantage of deep learning methods is that they can learn a task such as denoising from the data themselves, thus providing a sample-specific method that does not depend on a physical model. Once a network has been trained, subsequent image denoising using a convolutional neural network is fast compared to traditional methods, which are typically much slower.

Few datasets exist for evaluating fluorescence microscopy denoising. The dataset of Zhang et al. [23] contains 12,000 images captured with either a confocal, 2-photon, or wide-field microscope of various samples such as cells, zebrafish, and mouse brain tissues. They provide 50 low-SNR samples of each field of view (FOV), so that the high-SNR target can be recovered by averaging. However, they only provide 8-bit images and the quality of the images is limited. Similarly, Zhou et al. [24] provide 400 low-SNR samples of the same FOV over 120 different FOVs. Their data only provide wide-field images of human cells. Weigert et al. [17] evaluated denoising using a collection of image stacks including planaria, tribolium, flywing, *Drosophila*, retina, and liver samples. One drawback of this dataset is that each training split contains image patches, not whole images, which limits flexibility in the training set-up. Our dataset addresses the gaps in these previous datasets by providing whole images under both low-SNR and high-SNR exposure settings. Our dataset covers a wide range of sample types and imaging modalities, including wide-field images of cells in which actin, mitochondria, membrane, or nuclei are labeled, and confocal microscopy images of actin and mitochondria.

In addition to providing the datasets, we evaluated the performance of a recently proposed neural network for content-aware image restoration (CARE) of fluorescence microscopy images [17]. To do this we used CSBDeep [25], a toolbox for implementation of the CARE network. This network uses a series of convolutional layers from input to output in a U-Net architecture [26] and uses the mean squared error (MSE) loss function during training.

We also evaluated a self-supervised learning approach called Noise2Void (N2V) [27]. This method learns denoising using only the noisy data. It also uses a U-Net architecture and MSE loss function but masks out random pixels during training to force the network to learn to predict the denoised value of each masked pixel based on the neighborhood of that pixel in the noisy input. We used the reference implementation provided by the authors.

## Methods

### Fluorescence microscopy

We acquired datasets 1, 2, 3, 5, and 6 using an IX83 microscope equipped with UplanSApo 100×/1.40 numerical aperture (NA) oil immersion, 60×/1.35 NA oil immersion, and 20×/0.75 NA air objectives (Olympus, Tokyo, Japan), Zyla 4.2-plus sCMOS camera (Andor, Belfast, UK), and SpectraX light source (Lumencor, Beaverton, OR, USA). Focusing was achieved using a piezo-Z stage (Applied Scientific Instrumentation, Eugene, OR, USA). The system was controlled by IQ3 software (Andor). We used fluorescence filter set 59022 (Chroma, Bellows Falls, VT, USA). Dataset 4 was acquired with an SP5 laser scanning confocal microscope (Leica, Mannheim, Germany) using 488- and 543-nm lasers and an HCX PL APO CS 63×/1.4 NA oil immersion objective (Leica).

The sample in datasets 1–5 was a FluoCells No. 1 prepared slide (Molecular Probes, Eugene, OR, USA). This slide contains bovine pulmonary artery endothelial cells stained with MitoTracker Red CMXRos (labels mitochondria) and AlexaFluor 488 phalloidin (labels actin). The sample in dataset 6 was a HepG2 cell line that was grown on cover slips under standard conditions and labeled with the membrane probe DiI (Molecular Probes).

### Data analysis

In each dataset, the last 10% of images were used for testing and the remaining were used for training.

To train the CARE network, we used the following configuration. We used the ADAM optimizer [28], the training batch size was 16 images, the number of training epochs was 200, the initial learning rate was 0.0004, and the iterations per epoch (training steps) was 400. In sampling the training images, 800 patches per image of size 128 × 128 pixels were used to train the CARE network. In all experiments, 10% of the patches were withheld for validation during training, and the model with best validation error observed during training was saved and used for testing.

Following the standard implementation of the CARE network, we used the MSE loss function

(1) \begin{equation*}
\mathrm{MSE}\,\, = \frac{1}{{mn}}\,\,\mathop \sum \nolimits_{i\,\, = \,\,0}^{m - 1} \mathop \sum \nolimits_{j\,\, = \,\,0}^{n - 1} {\left[ {I\left( {i,j} \right) - K\left( {i,j} \right)} \right]^2}, \end{equation*}

where *I* is a high-SNR image, *K* is the corresponding low-SNR image after restoration, and *m* and *n* are the image height and width, respectively. This quantity is computed for each image in the batch and averaged to compute the loss. To train the N2V network, we used the same configuration as the CARE network but used the N2V training procedure. For comparison we used a standard denoising method, block matching and 3D filtering (BM3D) [29]. Following the procedure of Zhang et al. [23], on each image we estimated the noise level using the method of Foi et al. [30] and applied a variance-stabilizing transformation [31] before denoising the image with BM3D.

## Results

We acquired 6 datasets under different conditions. Table 1 provides an overview of the 6 datasets. In the wide-field data, we adjusted the camera exposure time such that the desired SNR levels were achieved. For confocal microscopy, we recorded images with 2 different (high or low) detector gains and laser powers.

**Table 1:** Overview of the datasets

Dataset	1 (60× Noise level 1)	2 (60× Noise level 2)	3 (20×)	4 (Confocal)	5 (Nucleus)	6 (Membrane)
Microscope	Widefield	Widefield	Widefield	Confocal	Widefield	Widefield
Objective	60 ×/1.35 NA	60 ×/1.35 NA	20 ×/0.75 NA	63 ×/1.4 NA	100×/1.40 NA	100×/1.40 NA
Pixel size, nm	108	108	325	96	65	65
Exposure times	(Actin) high exp 400 ms	(Actin) high exp 1,000 ms	(Actin) high exp 500 ms	laser 10%, det. gain 535 V	high exp 1,500 ms	high exp 450 ms
	(Actin) low exp 20 ms	(Actin) low exp 15 ms	(Actin) low exp 20 ms	laser 50% det. gain 619 V	low exp 40 ms	low exp 25 ms
	(Mito) high exp 400 ms	(Mito) high exp 600 ms	(Mito) high exp 400 ms	laser 20% det. gain 800 V		
	(Mito) low exp 20 ms	(Mito) low exp 10 ms	(Mito) low exp 15 ms	laser 66% det. gain 772 V		
Image size, pixels	2,048 × 2,048	2,048 × 2,048	2,048 × 2,048	1,024 × 1,024	512 × 512	2,048 × 2,048
No. of images	100	100	100	79	104	84

**Figure 1:**
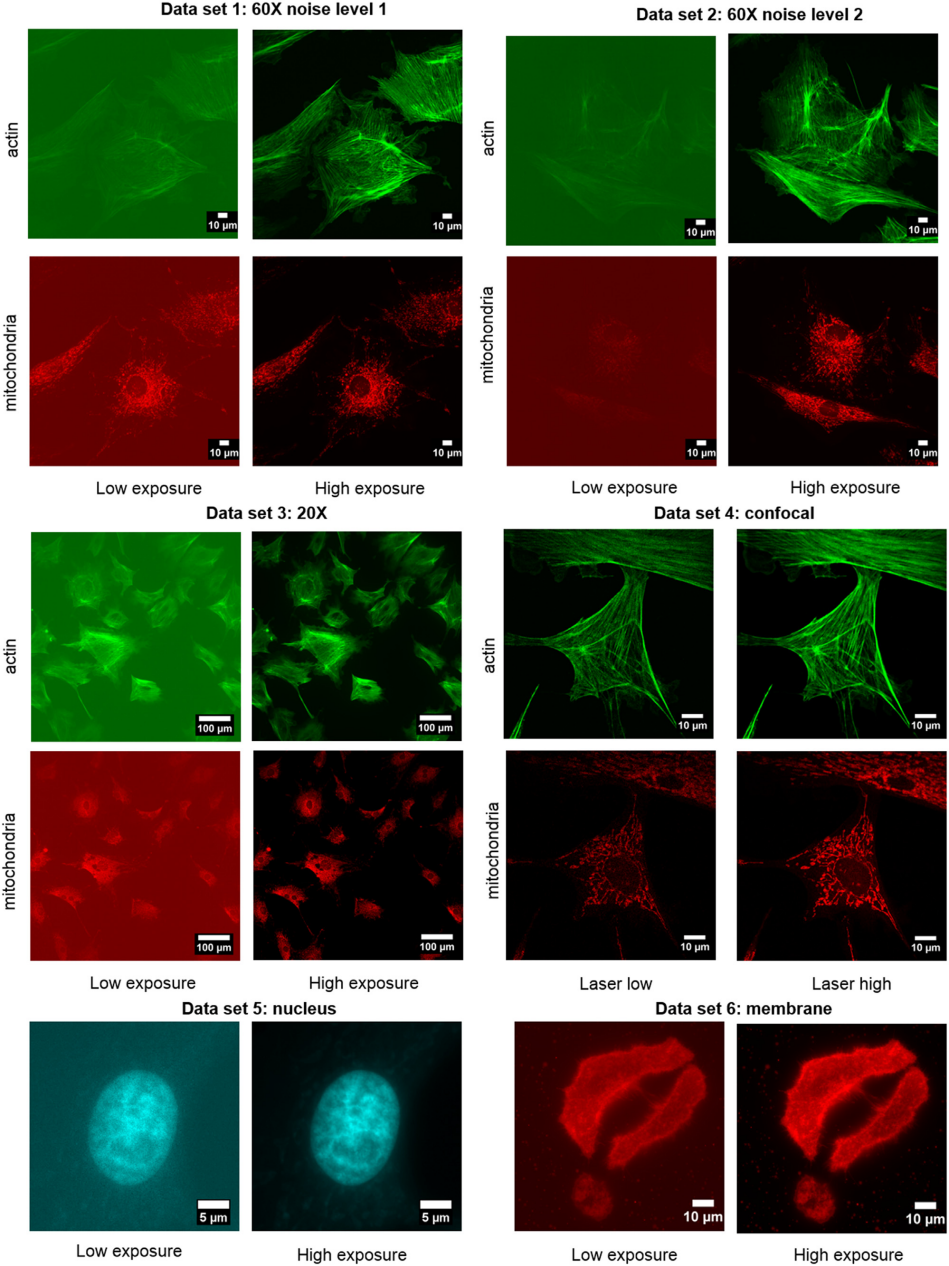
Example images from each of the 6 datasets.

Figure 1 shows examples images from each of the 6 datasets.

**Figure 2:**
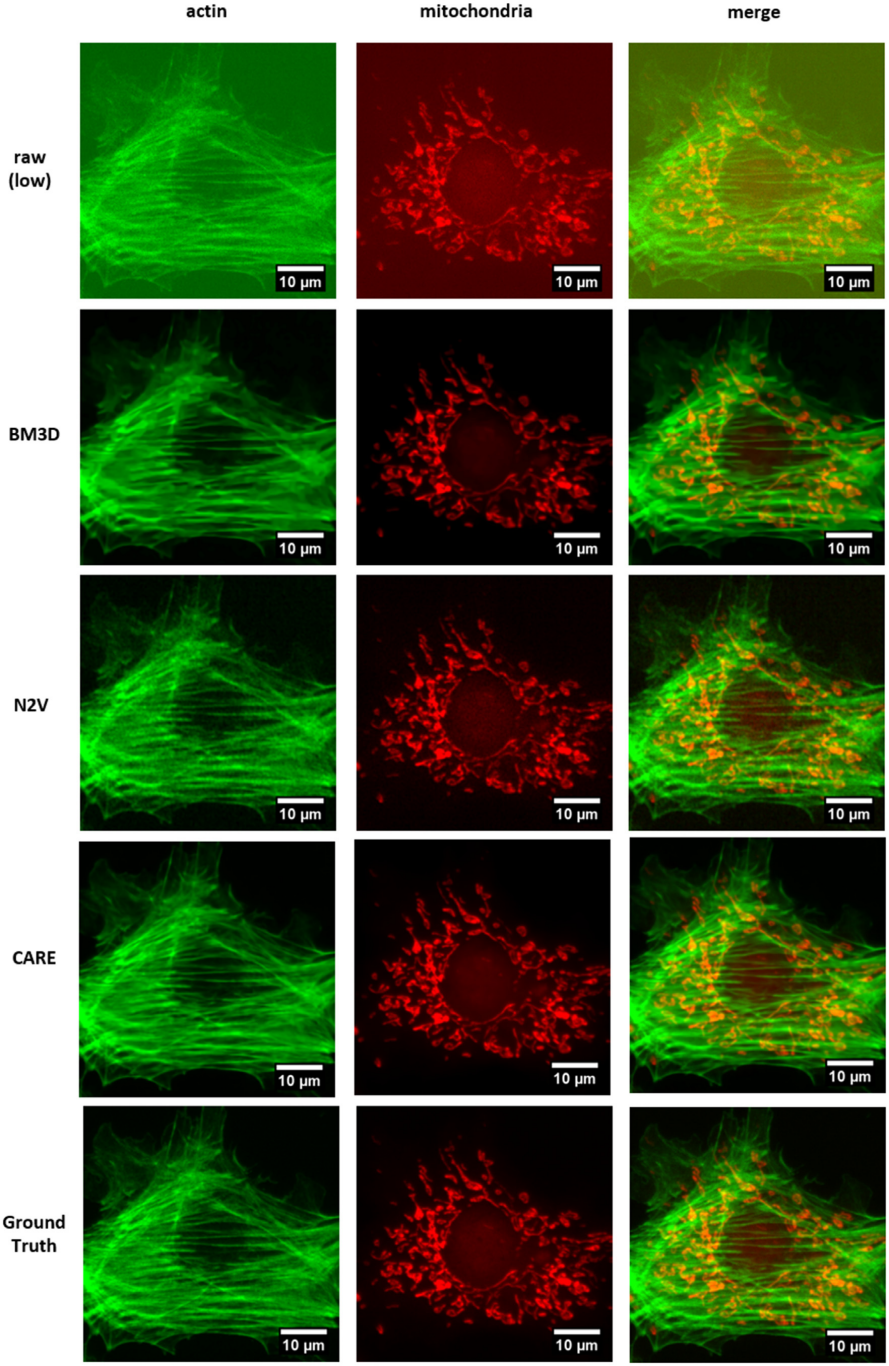
Results of denoising methods. Shown are selected images from dataset 1 (60× noise level 1).

After data acquisition, we tested 3 different methods for image denoising. Figure 2 shows the original low-exposure image (raw), the matching high-exposure image (ground truth), and the results of the CARE method, the N2V method, and a standard denoising method (BM3D). For this comparison we selected an image pair from dataset 1 (60× noise level 1).

Table 2 provides mean metrics for the denoising performance for each method on each dataset. We used 2 metrics: peak signal-to-noise ratio (PSNR) and structural similarity (SSIM). Before computing the metrics we scaled and shifted both images to minimize the MSE between them [17].

**Table 2:** Mean PSNR and SSIM results .

Dataset	PSNR, dB				SSIM			
	Raw (low)	CARE	N2V	BM3D	Original	CARE	N2V	BM3D
Actin 20×	24.10	32.12	29.26	30.35	0.37	0.90	0.74	0.87
Actin 60× (noise 1)	27.95	38.86	35.74	36.29	0.60	0.95	0.92	0.93
Actin 60× (noise 2)	18.34	28.89	22.77	25.16	0.09	0.81	0.32	0.53
Mito 20×	24.41	32.48	28.75	29.44	0.33	0.91	0.68	0.82
Mito 60× (noise 1)	27.91	39.30	33.80	36.70	0.55	0.97	0.87	0.94
Mito 60× (noise 2)	19.95	27.56	22.50	24.58	0.13	0.82	0.24	0.42
Actin Confocal	24.65	29.44	26.99	27.08	0.67	0.83	0.77	0.78
Mito Confocal	22.07	27.55	25.63	26.85	0.52	0.76	0.68	0.75
Nucleus	24.67	35.79	26.06	35.58	0.41	0.91	0.81	0.90
Membrane	29.40	35.35	29.52	35.09	0.64	0.93	0.83	0.93

Finally, the PSNR metric was calculated as

\begin{equation*} \mathrm{PSNR}\,\, = \,\,10\mathrm{lo}{\mathrm{g}_{10}}\left( {\frac{1}{{\mathrm{MSE}}}} \right). \end{equation*}

The SSIM metric [32] is an image quality metric designed to approximate human perception of similarity to a reference image. Unlike PSNR, the metric takes into account structural information in the image. The SSIM metric ranges from 0 to 1, with a greater number indicating higher quality.

As shown in Table 2, the unsupervised N2V method is the weakest performer on both metrics. BM3D is better on both metrics but surpassed by the supervised CARE method on all datasets. All methods exhibit a ∼10 dB drop in PSNR or greater on the noisier datasets (Noise 2) in comparison to Noise 1. Each method also performed ∼6–7 dB worse on 20× magnification data in comparison to the 60× magnification data.

Visual inspection of the restored images (example shown in Fig. 2) shows that, despite having high SSIM scores, the BM3D tends to blur the images more than the other methods. The results of the N2V method are noticeably noisier than the results of the other methods.

Table 3 presents a comparison of the methods in terms of computation time. Using a single Nvidia V100 GPU, the CARE network took ∼3.5 hours to train on a single dataset while the Noise2Void network took ∼3 hours. The CARE network took ∼1 second to process a single image while the Noise2Void network took approximately half that. The BM3D method does not require training but took 50 seconds to process a single image in MATLAB on a 2.6 GHz Intel Core i3-7100U processor.

**Table 3:** Training and processing times

Method	Training time (h)	Processing time for 1 image (s)
CARE	∼3.5	0.90
Noise2Void	∼3	0.39
BM3D		50

### Reuse potential

The provided data can be used to implement new methods in machine learning or to test modifications of existing approaches. The data can be used to evaluate methods for denoising, super-resolution, or generative modeling, as well as new image quality metrics, for example. The data could also be used to evaluate the generalizability of methods trained onone type of data and tested on another. High-quality, publicly available data of this type have been lacking.

## Data Availability

All raw and analyzed data underlying this article are available on GigaDB [33]. All files and data are distributed under the Creative Commons CC0 waiver, with a request for attribution.

## Abbreviations

BM3D: block matching and 3D filtering; CARE: content-aware image restoration; FOV: field of view; MSE: mean squared error; NA: numerical aperture; PSNR: peak signal-to-noise ratio; SNR: signal-to-noise ratio; SSIM: structural similarity index, N2V: Noise2Void.

## Competing Interests

The authors declare that they have no competing interests.

## Funding

This work was supported by the National Institutes of Health Grant No. 1R15GM128166-01. This work was also supported by the UCCS BioFrontiers center. The funding sources had no involvement in study design; in the collection, analysis, and interpretation of data; in the writing of the report; or in the decision to submit the article for publication. This material is based in part upon work supported by the National Science Foundation under Grant No. 1727033. Any opinions, findings, and conclusions or recommendations expressed in this material are those of the authors and do not necessarily reflect the views of the National Science Foundation.

## Authors' Contributions

T.A.N.: analyzed data; J.B.: acquired data; R.M.: acquired data; T.K.: analyzed data; J.V.: conceived project, analyzed data, supervised research, wrote the manuscript; G.M.H.: conceived project, acquired data, analyzed data, supervised research, wrote the manuscript.

## Supplementary Material

giab032_GIGA-D-20-00180_Original_Submission

giab032_GIGA-D-20-00180_Revision_1

giab032_Response_to_Reviewer_Comments_Original_Submission

giab032_Reviewer_1_Report_Original_Submission Chris Armit -- 7/15/2020 Reviewed

giab032_Reviewer_2_Report_Original_Submission Martin Weigert -- 7/23/2020 Reviewed

## References

[1] Icha J, Weber M, Waters JC, et al. Phototoxicity in live fluorescence microscopy, and how to avoid it. Bioessays. 2017;39(8):1700003. 10.1002/bies.201700003 28749075

[2] Dixit R, Cyr R. Cell damage and reactive oxygen species production induced by fluorescence microscopy: effect on mitosis and guidelines for non-invasive fluorescence microscopy. Plant J. 2003;36:280–90. 14535891 10.1046/j.1365-313x.2003.01868.x

[3] Bogdanov AM, Bogdanova EA, Chudakov DM, et al. Cell culture medium affects GFP photostability: A solution. Nat Methods. 2009;6:859–60. 19935837 10.1038/nmeth1209-859

[4] Bogdanov AM, Kudryavtseva EI, Lukyanov KA. Anti-fading media for live cell GFP imaging. PLoS One. 2012;7:e53004. 23285248 10.1371/journal.pone.0053004 PMC3528736

[5] Nishigaki T, Wood CD, Shiba K, et al. Stroboscopic illumination using light-emitting diodes reduces phototoxicity in fluorescence cell imaging. Biotechniques. 2006;41:191–7. 16925021 10.2144/000112220

[6] Hoebe RA, Van Oven CH, Gadella TWJ, et al. Controlled light-exposure microscopy reduces photobleaching and phototoxicity in fluorescence live-cell imaging. Nat Biotechnol. 2007;25:249–53. 17237770 10.1038/nbt1278

[7] Caarls W, Rieger B, De Vries AHB, et al. Minimizing light exposure with the programmable array microscope. J Microsc. 2010;101–10. 10.1111/j.1365-2818.2010.03413.x 21118211

[8] Arigovindan M, Fung JC, Elnatan D, et al. High-resolution restoration of 3D structures from widefield images with extreme low signal-to-noise-ratio. Proc Natl Acad Sci U S A. 2013;110:17344–9. 24106307 10.1073/pnas.1315675110 PMC3808589

[9] Sibarita JB . Deconvolution microscopy. Adv Biochem Eng Biotechnol. 2005;95:201–43. 16080270 10.1007/b102215

[10] Boulanger J, Kervrann C, Bouthemy P, et al. Patch-based nonlocal functional for denoising fluorescence microscopy image sequences. IEEE Trans Med Imaging. 2010;29:442–54. 19900849 10.1109/TMI.2009.2033991

[11] Soubies E, Soulez F, McCann MT, et al. Pocket guide to solve inverse problems with GlobalBioIm. Inverse Probl. 2019;35:104006.

[12] Verveer PJ, Gemkow MJ, Jovin TM. A comparison of image restoration approaches applied to three-dimensional confocal and wide-field fluorescence microscopy. J Microsc. 1999;193:50–61. 12558687 10.1046/j.1365-2818.1999.00421.x

[13] Setzer S, Steidl G, Teuber T. Deblurring Poissonian images by split Bregman techniques. J Vis Commun Image Represent. 2010;21:193–9.

[14] Lecun Y, Bengio Y, Hinton G. Deep learning. Nature. 2015;521:436–44. 26017442 10.1038/nature14539

[15] Ouyang W, Aristov A, Lelek M, et al. Deep learning massively accelerates super-resolution localization microscopy. Nat Biotechnol. 2018;36:460–8. 29658943 10.1038/nbt.4106

[16] Rivenson Y, Göröcs Z, Günaydin H, et al. Deep learning microscopy. Optica. 2017;4:1437.

[17] Weigert M, Schmidt U, Boothe T, et al. Content-aware image restoration: pushing the limits of fluorescence microscopy. Nat Methods. 2018;15:1090–7. 30478326 10.1038/s41592-018-0216-7

[18] Nehme E, Weiss LE, Michaeli T, et al. Deep-STORM: super-resolution single-molecule microscopy by deep learning. Optica. 2018;5:458.

[19] Zhang K, Zuo W, Chen Y, et al. Beyond a Gaussian denoiser: residual learning of deep CNN for image denoising. IEEE Trans Image Process. 2017;26:3142–55. 28166495 10.1109/TIP.2017.2662206

[20] Mao X-J, Shen C, Yang Y-B. Image restoration using convolutional auto-encoders with symmetric skip connections. arXiv. 2016:1606.08921.

[21] Khademi W, Rao S, Minnerath C, et al. Self-supervised Poisson-Gaussian denoising. In Proceedings of the IEEE/CVF Winter Conference on Applications of Computer Vision (WACV). 2021:2131–9. 10.1109/wacv48630.2021.00218 PMC8294668 34296053

[22] Rumelhart DE, Hinton GE, Williams RJ. Learning representations by back-propagating errors. Nature. 1986;323:533–6.

[23] Zhang Y, Zhu Y, Nichols E, et al. A Poisson-Gaussian denoising dataset with real fluorescence microscopy images. In Proceedings of the IEEE Computer Society Conference on Computer Vision and Pattern Recognition. IEEE; 2019:11702–10.

[24] Zhou R, El Helou M, Sage D, et al. W2S: microscopy data with joint denoising and super-resolution for widefield to SIM mapping. In: Computer Vision – ECCV 2020 Workshops. ECCV 2020. Cham:Springer; 2020:474–91.

[25] CSBDeep. https://csbdeep.bioimagecomputing.com/.Accessed April 30, 2021.

[26] Falk T, Mai D, Bensch R, et al. U-Net: deep learning for cell counting, detection, and morphometry. Nat Methods. 2019;16;67–70. 30559429 10.1038/s41592-018-0261-2

[27] Krull A, Buchholz T-O, Jug F. Noise2Void-learning denoising from single noisy images. In Proceedings of the IEEE Conference on Computer Vision and Pattern Recognition. 2019:2129–37.

[28] Kingma DP, Ba JL. ADAM: a method for stochastic optimization. AIP Conf Proc. 2014;1631:58–62.

[29] Dabov K, Foi A, Katkovnik V, et al. Image denoising with block-matching and 3D filtering. Proc SPIE. 2006;6064:606414.

[30] Foi A, Trimeche M, Katkovnik V, et al. Practical Poissonian-Gaussian noise modeling and fitting for single-image raw-data. IEEE Trans Image Process. 2008;17:1737–54. 18784024 10.1109/TIP.2008.2001399

[31] Mäkitalo M, Foi A. Optimal inversion of the generalized anscombe transformation for Poisson-Gaussian noise. IEEE Trans Image Process. 2013;22:91–103. 22692910 10.1109/TIP.2012.2202675

[32] Wang Z, Bovik AC, Sheikh HR, et al. Image quality assessment: From error visibility to structural similarity. IEEE Trans Image Process. 2004;13:600–12. 15376593 10.1109/tip.2003.819861

[33] Hagen GM, Bendesky J, Machado R, et al. Supporting data for “Fluorescence microscopy datasets for training deep neural networks.”. GigaScience Database. 2021. 10.5524/100888. PMC8099770 33954794

