# Supplementary material for: Fluorescence microscopy datasets for training deep neural networks
Source: Gigascience. 2021 May 5;10(5):giab032. doi: 10.1093/gigascience/giab032 (PMC8099770; doi:10.1093/gigascience/giab032)
Supplement: giab032_GIGA-D-20-00180_Revision_1 [file giab032_giga-d-20-00180_revision_1.pdf]

GigaScience

Fluorescence Microscopy Datasets for Training Deep Neural Networks

--Manuscript Draft--

**Manuscript Number:**	GIGA-D-20-00180R1
**Full Title:**	Fluorescence Microscopy Datasets for Training Deep Neural Networks
**Article Type:**	Data Note
**Funding Information:**	National Institute of General Medical Sciences (1R15GM128166-01) — Dr Guy M Hagen<br>Directorate for Biological Sciences (1727033) — Dr Guy M Hagen<br>BioFrontiers Institute, University of Colorado Colorado Springs — Dr Guy M Hagen

Abstract:

Background

Fluorescence microscopy is an important technique in many areas of biological research. Two factors which limit the usefulness and performance of fluorescence microscopy are photobleaching of fluorescent probes during imaging, and, when imaging live cells, phototoxicity caused by light exposure. Recently developed methods in machine learning are able to greatly improve the signal to noise ratio of acquired images. This allows researchers to record images with much shorter exposure times, which in turn minimizes photobleaching and phototoxicity by reducing the dose of light reaching the sample.

Findings

To employ deep learning methods, a large amount of data is needed to train the underlying convolutional neural network. One way to do this involves use of pairs of fluorescence microscopy images acquired with long and short exposure times. We provide high quality data sets which can be used to train and evaluate deep learning methods under development.

Conclusion

The availability of high quality data is vital for training convolutional neural networks which are used in current machine learning approaches.

**Corresponding Author:**	Guy M Hagen, PhD<br>University of Colorado Colorado Springs<br>Colorado Springs, CO UNITED STATES
**Corresponding Author Secondary Information:**	
**Corresponding Author's Institution:**	University of Colorado Colorado Springs
**Corresponding Author's Secondary Institution:**	
**First Author:**	Guy M Hagen, PhD
**First Author Secondary Information:**	
**Order of Authors:**	Guy M Hagen, PhD
	Justin Bendesky
	Rosa Machado
	Tram-Anh Nguyen
	Tanmay Kumar
	Jonathan Ventura, PhD

Powered by Editorial Manager® and ProduXion Manager® from Aries Systems Corporation

Order of Authors Secondary Information:	
Response to Reviewers:	Dear Editor,<br>We would like to submit a revised version of our manuscript “Fluorescence Microscopy Datasets for Training Deep Neural Networks” for consideration as a data note in GigaScience. We would like to thank the editors for your patience as we completed the revisions. We have made numerous changes in an effort to respond to all of the reviewer’s comments. Our thanks also go to the reviewers for your helpful suggestions. We would like to respond to the reviewers with the following changes and improvements to the paper.<br>Reviewer 1:<br>I see great reuse potential in these imaging datasets and this Data Note and supporting data should be considered for publication in the GigaScience "Digital Pathology - Translatable Datasets for Clinical Reuse and Machine Learning" Thematic Series.<br>Thank you for your comment about the reuse potential. We have already been contacted by a few researchers asking when the data would be available and so we anticipate that there will be continued interest in the paper and the data. We would like to have the paper be part of this thematic series if this option is still available.<br>To ensure reproducibility, I request that the authors submit to GigaDB the denoised image files generated by: 1) CSBDeep toolbox; 2) NVIDIA Self-Supervised Deep Image Denoising software; and 3) BM3D.<br>We have uploaded to GigaDB the denoised image files as requested. Please note that we switched from the NVIDIA self-supervised denoising network to the Noise2Void network as requested by reviewer 2. This is a similar unsupervised network for denoising.<br>Reviewer 2:<br>Publicly available training datasets for DL methods are an important driver of research, yet compared to other fields (as computer vision) such datasets are currently less commonly found for fluorescence microscopy. So I really like that the paper tries to make a contribution towards changing that situation. I similarly like that the authors compared results from several DL methods as well as a strong classical baseline that are used in practice.<br>Thank you for these encouraging comments about the paper and datasets.<br>1) The authors write that "High quality, publicly available data of this type has been lacking". However there are some datasets that provide this (e.g. for 3D denoising [17]). Additionally, there is a recent publication [A] that provides such a dataset for seemingly the exact same situation (mixed poisson gaussian noise, 2D fluorescence microscopy images) yet with more diverse images (BPAE cells being a subset of it) [A] Zhang et al. "A poisson-gaussian denoising dataset with real fluorescence microscopy images." CVPR. 2019. So I wonder how much different the proposed dataset (and DL models trained on it) would be compared to [A]?<br>The Zhang paper “A poisson-gaussian denoising dataset with real fluorescence microscopy images” is an excellent resource but the data offered there is limited in a couple of important ways. The authors collected 50 noisy samples of each image, then average these images to generate a ground truth image. This is not the same thing as collecting an image with a long exposure time as the ground truth. The images offered in the Zhang paper are 512x512 pixels while ours range from 512x512 in one dataset up to 2048x2048 in four of the datasets. Also the Zhang data is limited to 8 bits (of intensity information), while ours were recorded at 16 bit.<br>Furthermore, for a public dataset to be valuable, the distribution of training images has to have a certain heterogeneity, such that evaluation on that data serves as a robust assessment of any method. The proposed dataset however contains only images of the same fixed sample (endothelial cells) of two essentially very stereotypical structures (Actin filaments and mitochondria). This makes it very hard to use the dataset for training models to be applied on differing structures (e.g. nuclei, membranes).<br>We have expanded the paper and datasets to now include images of the cell nucleus and membrane as requested. There are now 6 total datasets, the properties of which are shown in table 1 of the paper.<br>2) The current way of presenting the dataset/images (i.e. the main contribution) is suboptimal. Including at least an overview figure with a representative image for each modality/noise level/structure would greatly improve the paper (I essentially had to download the whole dataset just to have a look at a single image for each dataset). Additionally, Figure 1 has severe visual glitches that make it impossible to inspect the

Powered by Editorial Manager® and ProduXion Manager® from Aries Systems Corporation

different denoising results. Finally, providing insets in the same figure for the denoised images would greatly help to see the differences of the compared methods.
We have included a new figure (now figure 1, the original figure 1 is now figure 2.) The new figure 1 shows example images and thereby an overview of the 6 datasets. We included an “examples” folder on the FTP site so that users can download a small portion of the total data and thus get a look at what the rest of the data would look like. Sorry about the severe problems with figure 1 in the PDF you downloaded. Please note that the PDF conversion used by the submission system badly reproduces images. Please click on the link on that page of the PDF document and you should be able to download the original high resolution PNG files.
- "Each dataset consisted of images of size 2048×2048 pixels" -> Apart from dataset 4?
We removed this and just stated that we acquired the datasets under different conditions, table 1 describes these conditions.
- The MSE formula on line 102 misses the lower limit in the sum ("j=0"?)
We corrected the formula.
- "Following the standard implementation of the CSBDeep network, we used the Laplacian loss function" -> The default loss function in CSBDeep is mean absolute error MAE without any probabilistic component (the config default is probabilistic=False). The laplace loss should only be used if the resulting probabilistic model is needed (e.g. when the additional confidence prediction might be useful), which for a normal denoising task is not the case. I therefore would suggest to rerun at least some of the experiments with the default setting (probabilistic=False) and see whether the results change.
We re-ran all of the data with the default settings.
- BlindSpot: “uses careful padding and cropping to force the network to…” -> Padding and cropping is not really the main distinction of Blindspot networks…..- The relatively poor performance of the BlindSpot Network seems to me a bit surprising. "We used our own implementation in Python using the Keras library" ->I think it would be more convincing when using one of the official implementations, e.g. https://github.com/juglab/n2v
We switched to the Noise2Void network using the official implementation as suggested.
- How was the parameter of BM3D (noise level sigma) tuned?
Following the procedure of [1], on each image we estimated the noise level using the method of Foi et al. [2] and applied a variance stabilizing transformation [3] before denoising the image with BM3D. This explanation was added to the paper.
- "We normalized both images by clipping values below the 1st percentile and above the 99th percentile". Doesn't this remove essential information of the image? What was the reason to clip?
That is a good point and we removed this unnecessary clipping in the new version of the experiments.
- What stopping criterion was used for the CARE/Blindspot network training?
We trained each network for 200 epochs. In all experiments, 10% of the patches were withheld for validation during training, and the model with best validation error observed during training was saved and used for testing. We visually inspected the loss curves and observed that the loss for each training run had converged.

We hope that with these changes the paper will now be acceptable for publication in GigaScience.

Sincerely,

Guy M. Hagen

1. Y. Zhang, Y. Zhu, E. Nichols, Q. Wang, S. Zhang, C. Smith, and S. Howard, "A poisson-gaussian denoising dataset with real fluorescence microscopy images," in

	Proceedings of the IEEE Computer Society Conference on Computer Vision and Pattern Recognition (IEEE, 2019), Vol. 2019-June, pp. 11702–11710.<br>2. A. Foi, M. Trimeche, V. Katkovnik, and K. Egiazarian, "Practical Poissonian-Gaussian noise modeling and fitting for single-image raw-data," IEEE Trans. Image Process. 17, 1737–1754 (2008).<br>3. M. Mäkitalo and A. Foi, "Optimal inversion of the generalized anscombe transformation for Poisson-Gaussian noise," IEEE Trans. Image Process. 22, 91–103 (2013).
**Additional Information:**	
**Question**	**Response**
Are you submitting this manuscript to a special series or article collection?	No
**Experimental design and statistics**<br><br>Full details of the experimental design and statistical methods used should be given in the Methods section, as detailed in our Minimum Standards Reporting Checklist. Information essential to interpreting the data presented should be made available in the figure legends.<br><br>Have you included all the information requested in your manuscript?	Yes
**Resources**<br><br>A description of all resources used, including antibodies, cell lines, animals and software tools, with enough information to allow them to be uniquely identified, should be included in the Methods section. Authors are strongly encouraged to cite Research Resource Identifiers (RRIDs) for antibodies, model organisms and tools, where possible.<br><br>Have you included the information requested as detailed in our Minimum Standards Reporting Checklist?	Yes
**Availability of data and materials**<br><br>All datasets and code on which the conclusions of the paper rely must be either included in your submission or	Yes

deposited in publicly available repositories (where available and ethically appropriate), referencing such data using a unique identifier in the references and in the "Availability of Data and Materials" section of your manuscript.<br><br>Have you have met the above requirement as detailed in our Minimum Standards Reporting Checklist?	

Powered by Editorial Manager® and ProduXion Manager® from Aries Systems Corporation

Manuscript with DOI

Click here to access/download;Manuscript;Hagen_revision_datasets for

Fluorescence Microscopy Datasets for Training Deep Neural Networks

Guy M. Hagen[1], Justin Bendesky[1], Rosa Machado[1], Tram-Anh Nguyen[2], Tanmay Kumar[3], Jonathan Ventura[3]

[1]UCCS BioFrontiers Center, University of Colorado at Colorado Springs, 1420 Austin Bluffs Parkway, Colorado Springs, Colorado, 80918.

[2]George Mason University, 4400 University Drive, Fairfax, Virginia, 22030.

[3]Department of Computer Science and Software Engineering, California Polytechnic State University, San Luis Obispo, California, 93407

Abstract

Background: Fluorescence microscopy is an important technique in many areas of biological research. Two factors which limit the usefulness and performance of fluorescence microscopy are photobleaching of fluorescent probes during imaging, and, when imaging live cells, phototoxicity caused by light exposure. Recently developed methods in machine learning are able to greatly improve the signal to noise ratio of acquired images. This allows researchers to record images with much shorter exposure times, which in turn minimizes photobleaching and phototoxicity by reducing the dose of light reaching the sample.

Findings: To employ deep learning methods, a large amount of data is needed to train the underlying convolutional neural network. One way to do this involves use of pairs of fluorescence microscopy images acquired with long and short exposure times. We provide high quality data sets which can be used to train and evaluate deep learning methods under development.

Conclusion: The availability of high quality data is vital for training convolutional neural networks which are used in current machine learning approaches.

Keywords: fluorescence microscopy, deep learning, convolutional neural networks

Data description

Context

Fluorescence microscopy is an important technique in many areas of biomedical research, but its use can be limited by photobleaching of fluorescent probe molecules caused by the excitation light which is used. In addition, reactive oxygen species which are generated by exposing samples to light can cause cell damage and even cell death, limiting imaging of live cells [1,2]. Many strategies have been devised to overcome this problem including the use of specialized culture media [3,4], pulsed excitation [5], or more elaborate methods such as controlled light exposure microscopy [6,7].

Another approach involves recording of fluorescence microscopy images with short exposure times, low excitation light intensity, or both. This results in images with low signal to noise ratios (SNRs), which can then be improved using a variety of image restoration approaches [8–12]. Noise in low light images of this type typically follows a Poisson-Gaussian distribution. This condition makes solving the inverse problem which arises in image restoration methods very difficult and has led to a number of approximate methods [13].

Recently, deep learning methods in artificial intelligence [14] have been applied to many problems in image analysis, including those in optical microscopy [15–18] and in image denoising [19–21]. Deep learning approaches typically require a large amount of data to train the underlying convolutional neural network [22], however, such datasets are not always available. Here we provide fluorescence microscopy datasets which can be used to train and evaluate neural networks for the purpose of image denoising. The dataset consists of pairs of images acquired with different exposure times (or in the case of confocal microscopy, different laser power and detector gain settings). After training, the network can subsequently be used to enhance the SNR of newly acquired images.

One advantage of deep learning methods is that they can learn a task such as denoising from the data itself, thus providing a sample-specific method which does not depend on a physical model. Once a network has

been trained, subsequent image denoising using a convolutional neural network is fast compared to traditional methods which are typically much slower.

Few datasets exist for evaluating fluorescence microscopy denoising. The dataset of Zhang et al. [23] contains 12,000 images captured with either a confocal, two-photon, or wide-field microscope of various samples such as cells, zebrafish, and mouse brain tissues. They provide fifty low-SNR samples of each field-of-view (FOV), so that the high-SNR target can be recovered by averaging. However, they only provide 8-bit images and the quality of the images is limited. Similarly, Zhou et al. [24] provide 400 low-SNR samples of the same FOV over 120 different FOVs. Their data only provides widefield images of human cells. Weigert et al. [17] evaluated denoising using a collection of image stacks including planaria, tribolium, flywing, *Drosophila*, retina, and liver samples. One drawback of this dataset is that each training split contains image patches, not whole images, which limits flexibility in the training setup. Our dataset addresses the gaps in these previous datasets by providing whole images under both low-SNR and high-SNR exposure settings. Our dataset covers a wide range of sample types and imaging modalities, including widefield images of cells in which actin, mitochondria, membrane, or nuclei are labeled, and confocal microscopy images of actin and mitochondria.

In addition to providing the datasets, we evaluated the performance of a recently proposed neural network for content-aware image restoration (CARE) of fluorescence microscopy images [17]. To do this we used CSBDeep [25], a toolbox for implementation of the CARE network. This network uses a series of convolutional layers from input to output in a U-Net architecture [26] and uses the mean-squared-error (MSE) loss function during training.

We also evaluated a self-supervised learning approach called Noise2Void (N2V) [27]. This method learns denoising using only the noisy data. It also uses a U-Net architecture and MSE loss function but masks out random pixels during training to force the network to learn to predict the denoised value of each masked pixel based on the neighborhood of that pixel in the noisy input. We used the reference implementation provided by the authors.

Methods

Fluorescence Microscopy

We acquired datasets 1, 2, 3, 5, and 6 using an IX83 microscope equipped with UplanSApo 100×/1.40 NA oil immersion, 60×/1.35 NA oil immersion, and 20×/0.75 NA air objectives (Olympus, Tokyo, Japan), Zyla 4.2-plus sCMOS camera (Andor, Belfast, UK), and SpectraX light source (Lumencor, Beaverton, OR, USA). Focusing was achieved using a piezo-Z stage (Applied Scientific Instrumentation, Eugene, OR, USA). The system was controlled by IQ3 software (Andor). We used fluorescence filter set 59022 (Chroma, Bellows Falls, VT, USA). Dataset 4 was acquired with a SP5 laser scanning confocal microscope (Leica, Mannheim, Germany) using 488 nm and 543 nm lasers and a HCX PL APO CS 63×/1.4 NA oil immersion objective (Leica).

The sample in datasets 1 - 5 was a FluoCells #1 prepared slide (Molecular Probes, Eugene, OR, USA). This slide contains bovine pulmonary artery endothelial cells stained with MitoTracker Red CMXRos (labels mitochondria) and AlexaFluor 488 phalloidin (labels actin). The sample in dataset 6 was a HepG2 cell line which was grown on coverslips under standard conditions and labeled with the membrane probe DiI (Molecular Probes).

Data Analysis

In each dataset, the last 10% of images were used for testing and the remaining were used for training.

To train the CARE network, we used the following configuration. We used the ADAM optimizer [28], the training batch size was 16 images, the number of training epochs was 200, the initial learning rate was 0.0004, and the iterations per epoch (training steps) was 400. In sampling the training images, 800 patches per image of size 128x128 pixels were used to train the CARE network. In all experiments, 10% of the patches were withheld for validation during training, and the model with best validation error observed during training was saved and used for testing.

Following the standard implementation of the CARE network, we used the MSE loss function

$$MSE = \frac{1}{mn}\sum_{i=0}^{m-1}\sum_{j=0}^{n-1}[I(i,j) - K(i,j)]^2 \quad (1)$$

where *I* is a high SNR image, *K* is the corresponding low SNR image after restoration, and *m* and *n* are the image height and width, respectively. This quantity is computed for each image in the batch and averaged to compute the loss. To train the N2V network, we used the same configuration as the CARE network but used the N2V training procedure. For comparison we used a standard denoising method, block matching and 3D filtering (BM3D) [29]. Following the procedure of [23], on each image we estimated the noise level using the method of Foi et al. [30] and applied a variance stabilizing transformation [31] before denoising the image with BM3D.

Results

We acquired six datasets under different conditions. Table 1 provides an overview of the four datasets. In the widefield data, we used adjusted the camera exposure time such that the desired signal to noise levels were achieved. For confocal microscopy, we recorded images with two different (high or low) detector gains and laser powers.

Table 1: Overview of the datasets

**Dataset**	**1 (60× noise level 1)**	**2 (60× noise level 2)**	**3 (20×)**	**4 (confocal)**
**Microscope**	Widefield	Widefield	Widefield	Confocal
**Objective**	60×/1.35 NA oil immersion	60×/1.35 NA oil immersion	20×/0.75 NA air	63×/1.4 NA oil immersion
**Pixel size**	108 nm	108 nm	325 nm	96 nm
**Exposure times**	high exposure (actin): 400 ms	high exposure (actin): 1000 ms	high exposure (actin): 500 ms	(actin) 488 nm laser 10%, det. gain 535 V
	low exposure (actin): 20 ms	low exposure (actin): 15 ms	low exposure (actin): 20 ms	(actin) 488 nm laser 50% det. gain 619 V
	high exposure (mito): 400 ms	high exposure (mito): 600 ms	high exposure (mito): 400 ms	(mito) 543 nm laser 20%

				det. gain 800 V
	low exposure (mito): 20 ms	low exposure (mito): 10 ms	low exposure (mito): 15 ms	(mito) 543 nm laser 66%<br>det. gain 772 V
**Image size, pixels**	2048 × 2048	2048 × 2048	2048 × 2048	1024 × 1024
**Num. of images**	100	100	100	79

**Data set**	**5 (nucleus)**	**6 (membrane)**
**Microscope**	Widefield	Widefield
**Objective**	100×/1.40NA oil immersion	100×/1.40NA oil immersion
**Pixel size**	65 nm	65 nm
**Exposure times**	high exposure: 1500 ms	high exposure: 450 ms
	low exposure: 40 ms	low exposure: 25 ms
**Image size, pixels**	512 × 512	2048 × 2048
**Num. of images**	104	84

Figure 1 shows examples images from each of the six datasets.

INSERT FIGURE 1

Figure 1: Example images from each of the six datasets.

After data acquisition, we tested three different methods for image denoising. Figure 2 shows the original low exposure image (raw), the matching high exposure image (ground truth), and the results of the CARE method, the N2V method, and a standard denoising method (BM3D). For this comparison we selected an image pair from data set 1 (60X noise level 1).

INSERT FIGURE 2

Figure 2: Results of denoising methods. Shown are selected images from dataset 1 (60X noise 1).

Table 2 provides average metrics for the denoising performance for each method on each dataset. We used two metrics: peak signal-to-noise ratio (PSNR) and structural similarity (SSIM). Before computing the metrics we scaled and shifted both images to minimize the mean squared error (MSE) between them [17].

Finally, the PSNR metric was calculated as

$$PSNR = 10log_{10}\left(\frac{1}{MSE}\right)$$

The SSIM metric [32] is an image quality metric designed to approximate human perception of similarity to a reference image. Unlike PSNR, the metric takes into account structural information in the image. The SSIM metric ranges from 0 to 1 with a greater number indicating higher quality.

As shown in Table 2, the unsupervised N2V method is the weakest performer on both metrics. BM3D is better on both metrics but surpassed by the supervised CARE method on almost all datasets. All methods exhibit an approximately 10 dB drop in PSNR or greater on the noisier datasets (Noise 2) in comparison to Noise 1. Each method also performed about 6-7 dB worse on 20× magnification data in comparison to the 60× magnification data.

Visual inspection of the restored images (example shown in Figure 1) shows that, despite having high SSIM scores, the BM3D tends to blur the images more than the other methods. The results of the N2V method are noticeably noisier than the results of the other methods.

Table 2. Average PSNR and SSIM results

	**PSNR, dB**				**SSIM**			
**Dataset**	**Raw (low)**	**CARE**	**N2V**	**BM3D**	**Original**	**CARE**	**N2V**	**BM3D**
**Actin 20x**	24.10	32.12	29.26	30.35	0.37	0.90	0.74	0.87
**Actin 60x (noise 1)**	27.95	38.86	35.74	36.29	0.60	0.95	0.92	0.93
**Actin 60x (noise 2)**	18.34	28.89	22.77	25.16	0.09	0.81	0.32	0.53
**Mito 20x**	24.41	32.48	28.75	29.44	0.33	0.91	0.68	0.82
**Mito 60x (noise 1)**	27.91	39.30	33.80	36.70	0.55	0.97	0.87	0.94
**Mito 60x (noise 2)**	19.95	27.56	22.50	24.58	0.13	0.82	0.24	0.42

**Actin Confocal**	24.65	29.44	26.99	27.08	0.67	0.83	0.77	0.78
**Mito Confocal**	22.07	27.55	25.63	26.85	0.52	0.76	0.68	0.75
**Nucleus**	24.67	35.79	26.06	35.58	0.41	0.91	0.81	0.90
**Membrane**	29.40	35.35	29.52	35.09	0.64	0.93	0.83	0.93

Table 3 presents an comparison of the methods in terms of computation time. Using a single Nvidia V100 GPU, the CARE network took about 3.5 hours to train on a single dataset while the Noise2Void network took about 3 hours. The CARE network took about 1 second to process a single image while the Noise2Void network took about half that. The BM3D method does not require training but took about 50 seconds to process a single image in MATLAB on a 2.6 GHz Intel Core i3-7100U processor.

Table 3. Training and processing times

	**Training time**	**Processing time for one image**
**CARE**	~3.5 hours	0.90 s
**Noise2Void**	~3 hours	0.39 s
**BM3D**	-	50 s

Reuse potential

The provided data can be used to implement new methods in machine learning or to test modifications of existing approaches. The data can be used to evaluate methods for denoising, super-resolution, or generative modeling, as well as new image quality metrics, for example. The data could also be used to evaluate the generalization ability of methods trained on one type of data and tested on another. High quality, publicly available data of this type has been lacking.

Availability of supporting data

All raw and analyzed data is available on GigaDB at [33]. All files and data are distributed under the Creative Commons CC0 waiver, with a request for attribution.

Abbreviations

SSIM: structural similarity index

PSNR: peak signal to noise ratio

NA: numerical aperture

Ethics approval and consent to participate

Not applicable

Consent for publication

Not applicable

Competing interests

The authors declare that they have no competing interests.

Funding

This work was supported by the National Institutes of Health grant number 1R15GM128166-01. This work was also supported by the UCCS BioFrontiers center. The funding sources had no involvement in study design; in the collection, analysis and interpretation of data; in the writing of the report; or in the decision to submit the article for publication. This material is based in part upon work supported by the National Science Foundation under Grant Number 1727033. Any opinions, findings, and conclusions or recommendations expressed in this material are those of the authors and do not necessarily reflect the views of the National Science Foundation.

Author Contributions

TN: analyzed data

JB: acquired data

RM: acquired data

TK: analyzed data

JV: conceived project, analyzed data, supervised research, wrote the paper

GH: conceived project, acquired data, analyzed data, supervised research, wrote the paper

References

1. J. Icha, M. Weber, J. C. Waters, and C. Norden, "Phototoxicity in live fluorescence microscopy, and how to avoid it," BioEssays **39**, 1700003 (2017).

2. R. Dixit and R. Cyr, "Cell damage and reactive oxygen species production induced by fluorescence microscopy: effect on mitosis and guidelines for non-invasive fluorescence microscopy," Plant J. **36**, 280–290 (2003).

3. A. M. Bogdanov, E. A. Bogdanova, D. M. Chudakov, T. V Gorodnicheva, S. Lukyanov, and K. A. Lukyanov, "Cell culture medium affects GFP photostability: A solution," Nat. Methods **6**, 859–860 (2009).

4. A. M. Bogdanov, E. I. Kudryavtseva, and K. A. Lukyanov, "Anti-Fading Media for Live Cell GFP Imaging," PLoS One **7**, e53004 (2012).

5. T. Nishigaki, C. D. Wood, K. Shiba, S. A. Baba, and A. Darszon, "Stroboscopic illumination using light-emitting diodes reduces phototoxicity in fluorescence cell imaging," Biotechniques **41**, 191–197 (2006).

6. R. A. Hoebe, C. H. Van Oven, T. W. J. Gadella, P. B. Dhonukshe, C. J. F. Van Noorden, and E.

M. M. Manders, "Controlled light-exposure microscopy reduces photobleaching and phototoxicity in fluorescence live-cell imaging," Nat. Biotechnolgy **25**, 249–253 (2007).

7. W. Caarls, B. Rieger, A. H. B. De Vries, D. J. Arndt-Jovin, and T. M. Jovin, "Minimizing light exposure with the programmable array microscope," J. Microsc. 101–110 (2010).

8. M. Arigovindan, J. C. Fung, D. Elnatan, V. Mennella, Y.-H. M. Chan, M. Pollard, E. Branlund, J. W. Sedat, and D. A. Agard, "High-resolution restoration of 3D structures from widefield images with extreme low signal-to-noise-ratio," Proc. Natl. Acad. Sci. **110**, 17344–17349 (2013).

9. J. B. Sibarita, "Deconvolution microscopy," in *Advances in Biochemical Engineering/Biotechnology* (Springer, Berlin, Heidelberg, 2005), Vol. 95, pp. 201–243.

10. J. Boulanger, C. Kervrann, P. Bouthemy, P. Elbau, J.-B. Sibarita, and J. Salamero, "Patch-based nonlocal functional for denoising fluorescence microscopy image sequences.," IEEE Trans. Med. Imaging **29**, 442–54 (2010).

11. E. Soubies, F. Soulez, M. T. McCann, T. Pham, L. Donati, T. Debarre, D. Sage, and M. Unser, "Pocket guide to solve inverse problems with GlobalBioIm," Inverse Probl. **35**, 104006 (2019).

12. P. J. Verveer, M. J. Gemkow, and T. M. Jovin, "A comparison of image restoration approaches applied to three-dimensional confocal and wide-field fluorescence microscopy," J. Microsc. **193**, 50–61 (1999).

13. S. Setzer, G. Steidl, and T. Teuber, "Deblurring Poissonian images by split Bregman techniques," J. Vis. Commun. Image Represent. **21**, 193–199 (2010).

14. Y. Lecun, Y. Bengio, and G. Hinton, "Deep learning," Nature **521**, 436–444 (2015).

15. W. Ouyang, A. Aristov, M. Lelek, X. Hao, and C. Zimmer, "Deep learning massively accelerates super-resolution localization microscopy," Nat. Biotechnol. **36**, 460–468 (2018).

16. Y. Rivenson, Z. Göröcs, H. Günaydin, Y. Zhang, H. Wang, and A. Ozcan, "Deep learning microscopy," Optica **4**, 1437 (2017).

17. M. Weigert, U. Schmidt, T. Boothe, A. Müller, A. Dibrov, A. Jain, B. Wilhelm, D. Schmidt, C. Broaddus, S. Culley, M. Rocha-Martins, F. Segovia-Miranda, C. Norden, R. Henriques, M. Zerial, M. Solimena, J. Rink, P. Tomancak, L. Royer, F. Jug, and E. W. Myers, "Content-aware image restoration: pushing the limits of fluorescence microscopy," Nat. Methods **15**, 1090–1097 (2018).

18. E. Nehme, L. E. Weiss, T. Michaeli, and Y. Shechtman, "Deep-STORM: super-resolution single-molecule microscopy by deep learning," Optica **5**, 458 (2018).

19. K. Zhang, W. Zuo, Y. Chen, D. Meng, and L. Zhang, "Beyond a Gaussian Denoiser: Residual Learning of Deep CNN for Image Denoising," IEEE Trans. Image Process. **26**, 3142–3155 (2017).

20. X.-J. Mao, C. Shen, and Y.-B. Yang, "Image Restoration Using Convolutional Auto-encoders with Symmetric Skip Connections," (2016).

21. W. Khademi, S. Rao, C. Minnerath, G. Hagen, and J. Ventura, "Self-Supervised Poisson-Gaussian Denoising," in *Proceedings of the IEEE/CVF Winter Conference on Applications of Computer Vision (WACV)* (2021), pp. 2131–2139.

22. D. E. Rumelhart, G. E. Hinton, and R. J. Williams, "Learning representations by back-propagating errors," Nature **323**, 533–536 (1986).

23. Y. Zhang, Y. Zhu, E. Nichols, Q. Wang, S. Zhang, C. Smith, and S. Howard, "A poisson-gaussian denoising dataset with real fluorescence microscopy images," in *Proceedings of the IEEE Computer Society Conference on Computer Vision and Pattern Recognition* (IEEE, 2019), Vol. 2019-June, pp. 11702–11710.

24. R. Zhou, M. El Helou, D. Sage, T. Laroche, A. Seitz, and S. Süsstrunk, "W2S: Microscopy Data with Joint Denoising and Super-Resolution for Widefield to SIM Mapping," in *Computer Vision –*

ECCV 2020 Workshops. ECCV 2020. Lecture Notes in Computer Science (Springer, Cham, 2020), pp. 474–491.

25. "CSBDeep," https://csbdeep.bioimagecomputing.com/.

26. T. Falk, D. Mai, R. Bensch, Ö. Çiçek, A. Abdulkadir, Y. Marrakchi, A. Böhm, J. Deubner, Z. Jäckel, K. Seiwald, A. Dovzhenko, O. Tietz, C. Dal Bosco, S. Walsh, D. Saltukoglu, T. L. Tay, M. Prinz, K. Palme, M. Simons, I. Diester, T. Brox, and O. Ronneberger, "U-Net: deep learning for cell counting, detection, and morphometry," Nat. Methods **16**, 67–70 (2019).

27. A. Krull, T.-O. Buchholz, and F. Jug, "Noise2Void-Learning Denoising from Single Noisy Images," in *Proceedings of the IEEE Conference on Computer Vision and Pattern Recognition* (2019), pp. 2129–2137.

28. K. Diederik and J. L. Ba, "ADAM: A Method for Stochastic Optimization," in *AIP Conference Proceedings* (2014), Vol. 1631, pp. 58–62.

29. K. Dabov, A. Foi, V. Katkovnik, and K. Egiazarian, "Image denoising with block-matching and 3D filtering," in *Image Processing: Algorithms and Systems, Neural Networks, and Machine Learning* (SPIE, 2006), Vol. 6064, p. 606414.

30. A. Foi, M. Trimeche, V. Katkovnik, and K. Egiazarian, "Practical Poissonian-Gaussian noise modeling and fitting for single-image raw-data," IEEE Trans. Image Process. **17**, 1737–1754 (2008).

31. M. Mäkitalo and A. Foi, "Optimal inversion of the generalized anscombe transformation for Poisson-Gaussian noise," IEEE Trans. Image Process. **22**, 91–103 (2013).

32. Z. Wang, A. C. Bovik, H. R. Sheikh, and E. P. Simoncelli, "Image quality assessment: From error visibility to structural similarity," IEEE Trans. Image Process. **13**, 600–612 (2004).

33. Hagen GM; Bendesky J; Machado R; Nguyen T; Kumar T; Ventura J: Supporting data for

"Fluorescence Microscopy Datasets for Training Deep Neural Networks" GigaScience Database. 2021. http://dx.doi.org/10.5524/100888.

Figure1 Click here to access/download;Figure;figure 1.png

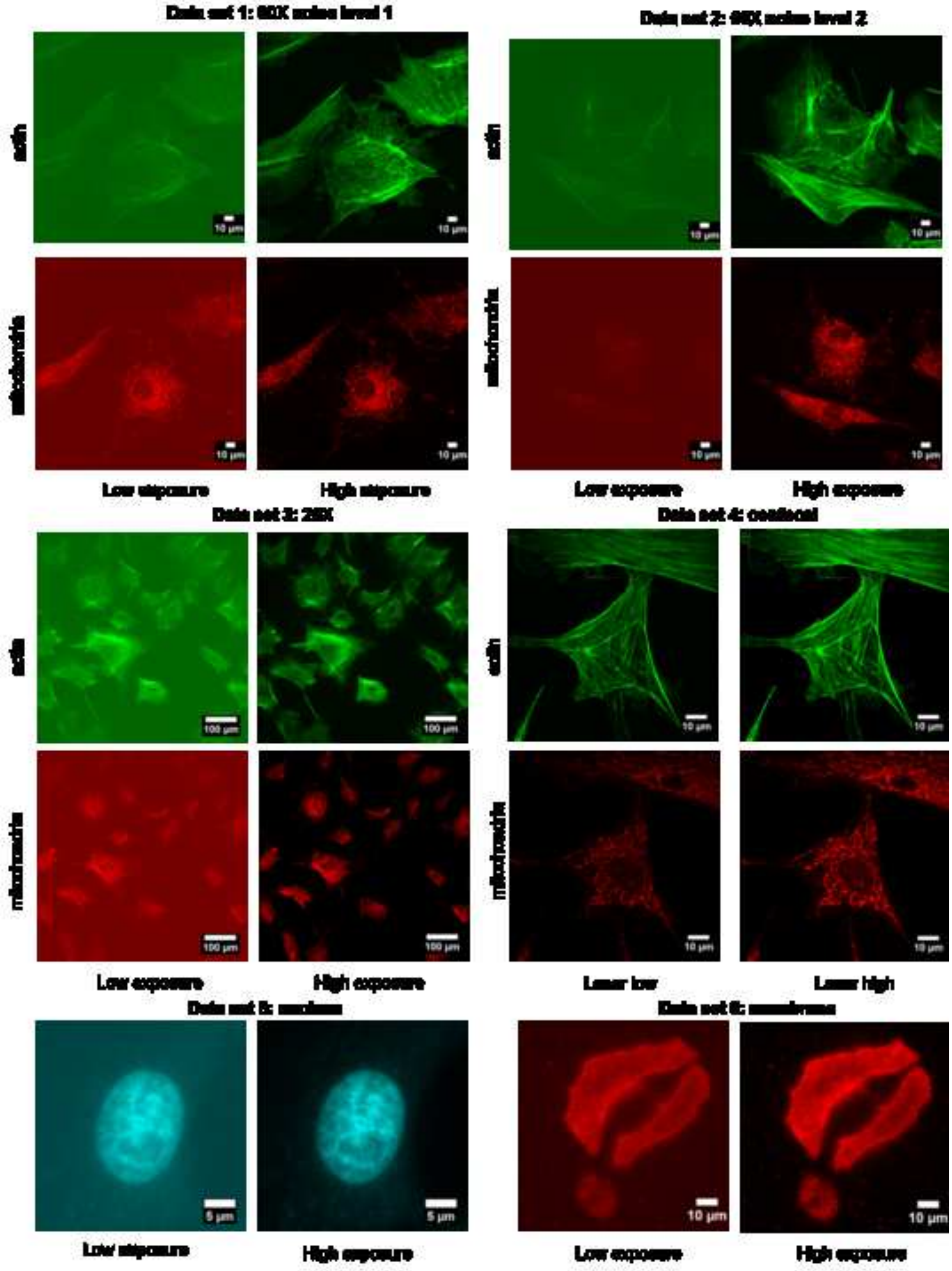

Figure2

Click here to access/download;Figure;figure 2.png

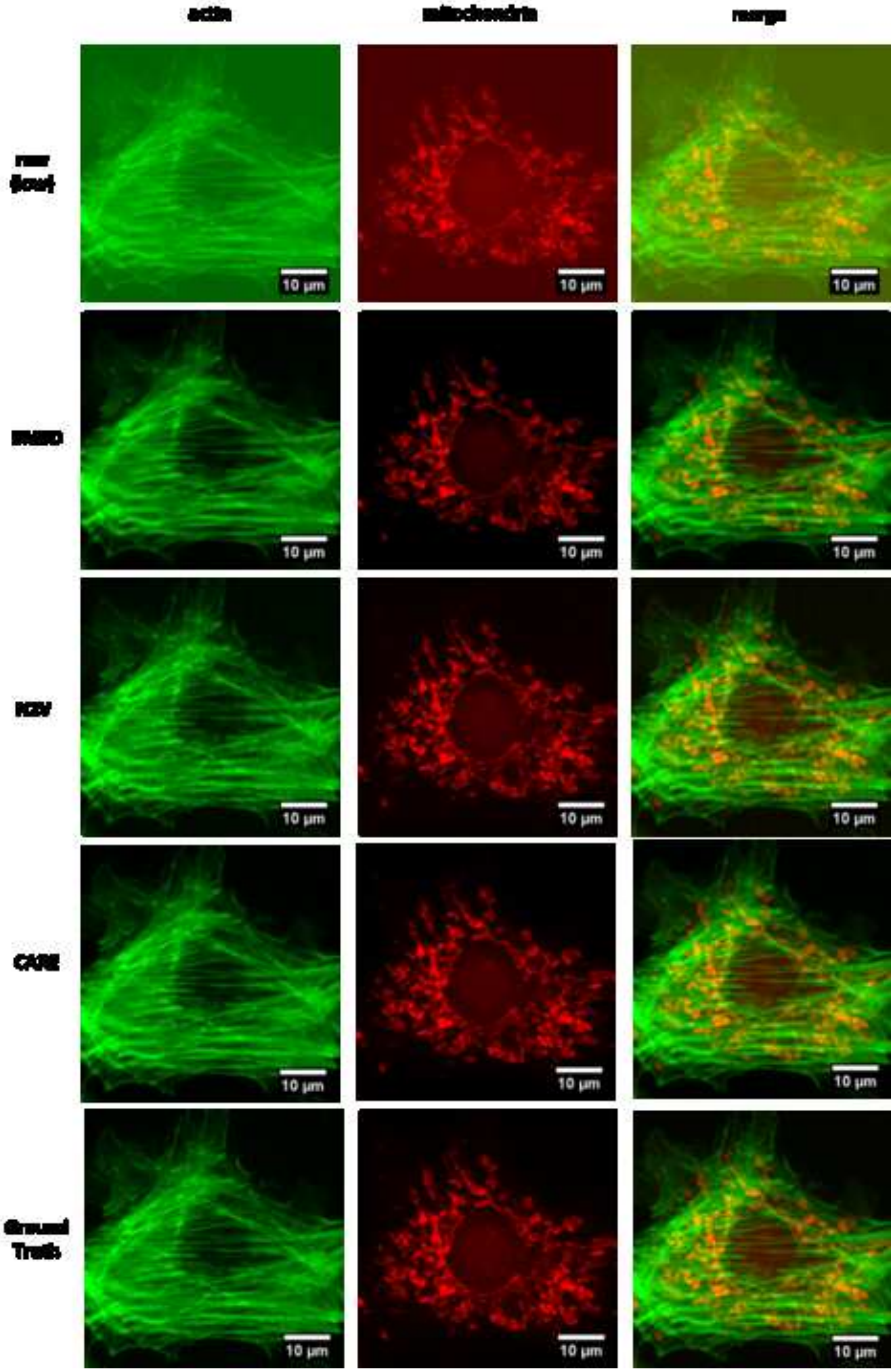

Personal Cover Click here to access/download;Personal Cover;Hagen letter to editor_revision.docx

University of Colorado Colorado Springs

Dr. Guy M. Hagen
Biofrontiers
1420 Austin Bluffs Pkwy.
Colorado Springs, CO 80918
Tel. 719-255-3692
ghagen@uccs.edu

Dear Editor,

We would like to submit a revised version of our manuscript "Fluorescence Microscopy Datasets for Training Deep Neural Networks" for consideration as a data note in *GigaScience.* We would like to thank the editors for your patience as we completed the revisions. We have made numerous changes in an effort to respond to all of the reviewer's comments. Our thanks also go to the reviewers for your helpful suggestions. We would like to respond to the reviewers with the following changes and improvements to the paper.

Reviewer 1:

I see great reuse potential in these imaging datasets and this Data Note and supporting data should be considered for publication in the GigaScience "Digital Pathology - Translatable Datasets for Clinical Reuse and Machine Learning" Thematic Series.

Thank you for your comment about the reuse potential. We have already been contacted by a few researchers asking when the data would be available and so we anticipate that there will be continued interest in the paper and the data. We would like to have the paper be part of this thematic series if this option is still available.

To ensure reproducibility, I request that the authors submit to GigaDB the denoised image files generated by: 1) CSBDeep toolbox; 2) NVIDIA Self-Supervised Deep Image Denoising software; and 3) BM3D.

We have uploaded to GigaDB the denoised image files as requested. Please note that we switched from the NVIDIA self-supervised denoising network to the Noise2Void network as requested by reviewer 2. This is a similar unsupervised network for denoising.

Reviewer 2:

Publicly available training datasets for DL methods are an important driver of research, yet compared to other fields (as computer vision) such datasets are currently less commonly found for fluorescence microscopy. So I really like that the paper tries to make a contribution towards changing that situation. I similarly like that the authors compared results from several DL methods as well as a strong classical baseline that are used in practice.

Thank you for these encouraging comments about the paper and datasets.

1) The authors write that "High quality, publicly available data of this type has been lacking". However there are some datasets that provide this (e.g. for 3D denoising [17]). Additionally, there is a recent publication [A] that provides such a dataset for seemingly the exact same situation (mixed poisson gaussian noise, 2D fluorescence microscopy images) yet with more diverse images (BPAE cells being a subset of it) [A] Zhang et al. "A poisson-gaussian denoising dataset with real fluorescence microscopy

University of Colorado Colorado Springs

Dr. Guy M. Hagen
Biofrontiers
1420 Austin Bluffs Pkwy.
Colorado Springs, CO 80918
Tel. 719-255-3692
ghagen@uccs.edu

images." CVPR. 2019. So I wonder how much different the proposed dataset (and DL models trained on it) would be compared to [A]?

The Zhang paper “A poisson-gaussian denoising dataset with real fluorescence microscopy images” is an excellent resource but the data offered there is limited in a couple of important ways. The authors collected 50 noisy samples of each image, then average these images to generate a ground truth image. This is not the same thing as collecting an image with a long exposure time as the ground truth. The images offered in the Zhang paper are 512x512 pixels while ours range from 512x512 in one dataset up to 2048x2048 in four of the datasets. Also the Zhang data is limited to 8 bits (of intensity information), while ours were recorded at 16 bit.

Furthermore, for a public dataset to be valuable, the distribution of training images has to have a certain heterogeneity, such that evaluation on that data serves as a robust assessment of any method. The proposed dataset however contains only images of the same fixed sample (endothelial cells) of two essentially very stereotypical structures (Actin filaments and mitochondria). This makes it very hard to use the dataset for training models to be applied on differing structures (e.g. nuclei, membranes).

We have expanded the paper and datasets to now include images of the cell nucleus and membrane as requested. There are now 6 total datasets, the properties of which are shown in table 1 of the paper.

2) The current way of presenting the dataset/images (i.e. the main contribution) is suboptimal. Including at least an overview figure with a representative image for each modality/noise level/structure would greatly improve the paper (I essentially had to download the whole dataset just to have a look at a single image for each dataset). Additionally, Figure 1 has severe visual glitches that make it impossible to inspect the different denoising results. Finally, providing insets in the same figure for the denoised images would greatly help to see the differences of the compared methods.

We have included a new figure (now figure 1, the original figure 1 is now figure 2.) The new figure 1 shows example images and thereby an overview of the 6 datasets. We included an “examples” folder on the FTP site so that users can download a small portion of the total data and thus get a look at what the rest of the data would look like.

Sorry about the severe problems with figure 1 in the PDF you downloaded. Please note that the PDF conversion used by the submission system badly reproduces images. Please click on the link on that page of the PDF document and you should be able to download the original high resolution PNG files.

- "Each dataset consisted of images of size 2048×2048 pixels" -> Apart from dataset 4?

We removed this and just stated that we acquired the datasets under different conditions, table 1 describes these conditions.

- The MSE formula on line 102 misses the lower limit in the sum ("j=0"?)

University of Colorado Colorado Springs

Dr. Guy M. Hagen
Biofrontiers
1420 Austin Bluffs Pkwy.
Colorado Springs, CO 80918
Tel. 719-255-3692
ghagen@uccs.edu

We corrected the formula.

- "Following the standard implementation of the CSBDeep network, we used the Laplacian loss function" -> The default loss function in CSBDeep is mean absolute error MAE without any probabilistic component (the config default is probabilistic=False). The laplace loss should only be used if the resulting probabilistic model is needed (e.g. when the additional confidence prediction might be useful), which for a normal denoising task is not the case. I therefore would suggest to rerun at least some of the experiments with the default setting (probabilistic=False) and see whether the results change.

We re-ran all of the data with the default settings.

- BlindSpot: "uses careful padding and cropping to force the network to…" -> Padding and cropping is not really the main distinction of Blindspot networks…..- The relatively poor performance of the BlindSpot Network seems to me a bit surprising. "We used our own implementation in Python using the Keras library" ->I think it would be more convincing when using one of the official implementations, e.g. https://github.com/juglab/n2v

We switched to the Noise2Void network using the official implementation as suggested.

- How was the parameter of BM3D (noise level sigma) tuned?

Following the procedure of [1], on each image we estimated the noise level using the method of Foi et al. [2] and applied a variance stabilizing transformation [3] before denoising the image with BM3D. This explanation was added to the paper.

- "We normalized both images by clipping values below the 1st percentile and above the 99th percentile". Doesn't this remove essential information of the image? What was the reason to clip?

That is a good point and we removed this unnecessary clipping in the new version of the experiments.

- What stopping criterion was used for the CARE/Blindspot network training?

We trained each network for 200 epochs. In all experiments, 10% of the patches were withheld for validation during training, and the model with best validation error observed during training was saved and used for testing. We visually inspected the loss curves and observed that the loss for each training run had converged.

University of Colorado
Colorado Springs

Dr. Guy M. Hagen
Biofrontiers
1420 Austin Bluffs Pkwy.
Colorado Springs, CO 80918
Tel. 719-255-3692
ghagen@uccs.edu

We hope that with these changes the paper will now be acceptable for publication in *GigaScience.*

Sincerely,

Guy Hagen

Guy M. Hagen

1. Y. Zhang, Y. Zhu, E. Nichols, Q. Wang, S. Zhang, C. Smith, and S. Howard, "A poisson-gaussian denoising dataset with real fluorescence microscopy images," in *Proceedings of the IEEE Computer Society Conference on Computer Vision and Pattern Recognition* (IEEE, 2019), Vol. 2019-June, pp. 11702–11710.

2. A. Foi, M. Trimeche, V. Katkovnik, and K. Egiazarian, "Practical Poissonian-Gaussian noise modeling and fitting for single-image raw-data," IEEE Trans. Image Process. **17**, 1737–1754 (2008).

3. M. Mäkitalo and A. Foi, "Optimal inversion of the generalized anscombe transformation for Poisson-Gaussian noise," IEEE Trans. Image Process. **22**, 91–103 (2013).
