# Supplementary material for: Fluorescence microscopy datasets for training deep neural networks
Source: Gigascience. 2021 May 5;10(5):giab032. doi: 10.1093/gigascience/giab032 (PMC8099770; doi:10.1093/gigascience/giab032)
Supplement: giab032_Response_to_Reviewer_Comments_Original_Submission [file giab032_response_to_reviewer_comments_original_submission.pdf]

Author's Response To Reviewer Comments

Close

Dear Editor,
We would like to submit a revised version of our manuscript “Fluorescence Microscopy Datasets for Training Deep Neural Networks” for consideration as a data note in GigaScience. We would like to thank the editors for your patience as we completed the revisions. We have made numerous changes in an effort to respond to all of the reviewer’s comments. Our thanks also go to the reviewers for your helpful suggestions. We would like to respond to the reviewers with the following changes and improvements to the paper.
Reviewer 1:
I see great reuse potential in these imaging datasets and this Data Note and supporting data should be considered for publication in the GigaScience "Digital Pathology - Translatable Datasets for Clinical Reuse and Machine Learning" Thematic Series.
Thank you for your comment about the reuse potential. We have already been contacted by a few researchers asking when the data would be available and so we anticipate that there will be continued interest in the paper and the data. We would like to have the paper be part of this thematic series if this option is still available.
To ensure reproducibility, I request that the authors submit to GigaDB the denoised image files generated by: 1) CSBDeep toolbox; 2) NVIDIA Self-Supervised Deep Image Denoising software; and 3) BM3D.
We have uploaded to GigaDB the denoised image files as requested. Please note that we switched from the NVIDIA self-supervised denoising network to the Noise2Void network as requested by reviewer 2. This is a similar unsupervised network for denoising.
Reviewer 2:
Publicly available training datasets for DL methods are an important driver of research, yet compared to other fields (as computer vision) such datasets are currently less commonly found for fluorescence microscopy. So I really like that the paper tries to make a contribution towards changing that situation. I similarly like that the authors compared results from several DL methods as well as a strong classical baseline that are used in practice.
Thank you for these encouraging comments about the paper and datasets.
1) The authors write that "High quality, publicly available data of this type has been lacking". However there are some datasets that provide this (e.g. for 3D denoising [17]). Additionally, there is a recent publication [A] that provides such a dataset for seemingly the exact same situation (mixed poisson gaussian noise, 2D fluorescence microscopy images) yet with more diverse images (BPAE cells being a subset of it) [A] Zhang et al. "A poisson-gaussian denoising dataset with real fluorescence microscopy images." CVPR. 2019. So I wonder how much different the proposed dataset (and DL models trained on it) would be compared to [A]?
The Zhang paper “A poisson-gaussian denoising dataset with real fluorescence microscopy images” is an excellent resource but the data offered there is limited in a couple of important ways. The authors collected 50 noisy samples of each image, then average these images to generate a ground truth image. This is not the same thing as collecting an image with a long exposure time as the ground truth. The images offered in the Zhang paper are 512x512 pixels while ours range from 512x512 in one dataset up to 2048x2048 in four of the datasets. Also the Zhang data is limited to 8 bits (of intensity information), while ours were recorded at 16 bit.
Furthermore, for a public dataset to be valuable, the distribution of training images has to have a certain heterogeneity, such that evaluation on that data serves as a robust assessment of any method. The proposed dataset however contains only images of the same fixed sample (endothelial cells) of two essentially very stereotypical structures (Actin filaments and mitochondria). This makes it very hard to use the dataset for training models to be applied on differing structures (e.g. nuclei, membranes).
We have expanded the paper and datasets to now include images of the cell nucleus and membrane as requested. There are now 6 total datasets, the properties of which are shown in table 1 of the paper.
2) The current way of presenting the dataset/images (i.e. the main contribution) is suboptimal. Including at least an overview figure with a representative image for each modality/noise level/structure would greatly improve the paper (I essentially had to download the whole dataset just to have a look at a single image for each dataset). Additionally, Figure 1 has severe visual glitches that make it impossible

to inspect the different denoising results. Finally, providing insets in the same figure for the denoised images would greatly help to see the differences of the compared methods.
We have included a new figure (now figure 1, the original figure 1 is now figure 2.) The new figure 1 shows example images and thereby an overview of the 6 datasets. We included an “examples” folder on the FTP site so that users can download a small portion of the total data and thus get a look at what the rest of the data would look like.
Sorry about the severe problems with figure 1 in the PDF you downloaded. Please note that the PDF conversion used by the submission system badly reproduces images. Please click on the link on that page of the PDF document and you should be able to download the original high resolution PNG files.
- "Each dataset consisted of images of size 2048×2048 pixels" -> Apart from dataset 4?
We removed this and just stated that we acquired the datasets under different conditions, table 1 describes these conditions.
- The MSE formula on line 102 misses the lower limit in the sum ("j=0"?)
We corrected the formula.
- "Following the standard implementation of the CSBDeep network, we used the Laplacian loss function" -> The default loss function in CSBDeep is mean absolute error MAE without any probabilistic component (the config default is probabilistic=False). The laplace loss should only be used if the resulting probabilistic model is needed (e.g. when the additional confidence prediction might be useful), which for a normal denoising task is not the case. I therefore would suggest to rerun at least some of the experiments with the default setting (probabilistic=False) and see whether the results change.
We re-ran all of the data with the default settings.
- BlindSpot: “uses careful padding and cropping to force the network to…” -> Padding and cropping is not really the main distinction of Blindspot networks…..- The relatively poor performance of the BlindSpot Network seems to me a bit surprising. "We used our own implementation in Python using the Keras library" ->I think it would be more convincing when using one of the official implementations, e.g. https://github.com/juglab/n2v
We switched to the Noise2Void network using the official implementation as suggested.
- How was the parameter of BM3D (noise level sigma) tuned?
Following the procedure of [1], on each image we estimated the noise level using the method of Foi et al. [2] and applied a variance stabilizing transformation [3] before denoising the image with BM3D. This explanation was added to the paper.
- "We normalized both images by clipping values below the 1st percentile and above the 99th percentile". Doesn't this remove essential information of the image? What was the reason to clip?
That is a good point and we removed this unnecessary clipping in the new version of the experiments.
- What stopping criterion was used for the CARE/Blindspot network training?
We trained each network for 200 epochs. In all experiments, 10% of the patches were withheld for validation during training, and the model with best validation error observed during training was saved and used for testing. We visually inspected the loss curves and observed that the loss for each training run had converged.

We hope that with these changes the paper will now be acceptable for publication in GigaScience.

Sincerely,

Guy M. Hagen

1. Y. Zhang, Y. Zhu, E. Nichols, Q. Wang, S. Zhang, C. Smith, and S. Howard, "A poisson-gaussian denoising dataset with real fluorescence microscopy images," in Proceedings of the IEEE Computer Society Conference on Computer Vision and Pattern Recognition (IEEE, 2019), Vol. 2019-June, pp. 11702–11710.
2. A. Foi, M. Trimeche, V. Katkovnik, and K. Egiazarian, "Practical Poissonian-Gaussian noise modeling and fitting for single-image raw-data," IEEE Trans. Image Process. 17, 1737–1754 (2008).
3. M. Mäkitalo and A. Foi, "Optimal inversion of the generalized anscombe transformation for Poisson-Gaussian noise," IEEE Trans. Image Process. 22, 91–103 (2013).

Close
