# Supplementary material for: Fluorescence microscopy datasets for training deep neural networks
Source: Gigascience. 2021 May 5;10(5):giab032. doi: 10.1093/gigascience/giab032 (PMC8099770; doi:10.1093/gigascience/giab032)
Supplement: giab032_Reviewer_1_Report_Original_Submission — Chris Armit -- 7/15/2020 Reviewed [file giab032_reviewer_1_report_original_submission.pdf]

Reviewer Report

Title: Fluorescence Microscopy Datasets for Training Deep Neural Networks

Version: Original Submission **Date:** 7/15/2020

Reviewer name: Chris Armit

Reviewer Comments to Author:

This Data Note describes widefield and confocal fluorescence microscopy imaging datasets that can be reused in the context of machine learning to explore different methods of improving the signal to noise ratio of acquired images. As the authors explain, "to employ deep learning methods, a large amount of data is needed to train the underlying convolutional neural network" and that "one way to do this involves use of pairs of fluorescence microscopy images acquired with long and short exposure times". Towards this end, the authors provide high quality imaging datasets of bovine pulmonary artery endothelial cells, stained with MitoTracker Red CMXRos - which labels mitochondria - and additionally stained with AlexaFluor 488 phalloidin, which labels actin. Each dataset consists of 100 pairs of images (widefield) or 79 pairs of images (confocal) acquired with different exposure times, and the authors showcase the utility of these imaging datasets by exploring denoising using an open source CSBDeep toolbox content-aware image restoration (CARE) neural network. The authors additionally explored denoising through use of a Self-Supervised Deep Image Denoising neural network that is under copyright by NVIDIA Corporation and that has been ascribed a Creative Commons BY-NC 4.0 license (https://github.com/NVlabs/selfsupervised-denoising). For comparison the authors used a standard denoising method, block matching and 3D filtering (BM3D).
I see great reuse potential in these imaging datasets and this Data Note and supporting data should be considered for publication in the GigaScience "Digital Pathology - Translatable Datasets for Clinical Reuse and Machine Learning" Thematic Series.
Minor comment
To ensure reproducibility, I request that the authors submit to GigaDB the denoised image files generated by: 1) CSBDeep toolbox; 2) NVIDIA Self-Supervised Deep Image Denoising software; and 3) BM3D.

Level of Interest

Please indicate how interesting you found the manuscript: Choose an item.

Quality of Written English

Please indicate the quality of language in the manuscript: Choose an item.

Declaration of Competing Interests

Please complete a declaration of competing interests, considering the following questions:

- Have you in the past five years received reimbursements, fees, funding, or salary from an organisation that may in any way gain or lose financially from the publication of this manuscript, either now or in the future?
- Do you hold any stocks or shares in an organisation that may in any way gain or lose financially from the publication of this manuscript, either now or in the future?
- Do you hold or are you currently applying for any patents relating to the content of the manuscript?
- Have you received reimbursements, fees, funding, or salary from an organization that holds or has applied for patents relating to the content of the manuscript?
- Do you have any other financial competing interests?
- Do you have any non-financial competing interests in relation to this paper?

If you can answer no to all of the above, write 'I declare that I have no competing interests' below. If your reply is yes to any, please give details below.

I declare that I have no competing interests.

I agree to the open peer review policy of the journal. I understand that my name will be included on my report to the authors and, if the manuscript is accepted for publication, my named report including any attachments I upload will be posted on the website along with the authors' responses. I agree for my report to be made available under an Open Access Creative Commons CC-BY license (http://creativecommons.org/licenses/by/4.0/). I understand that any comments which I do not wish to be included in my named report can be included as confidential comments to the editors, which will not be published.

Choose an item.

To further support our reviewers, we have joined with Publons, where you can gain additional credit to further highlight your hard work (see: https://publons.com/journal/530/gigascience). On publication of this paper, your review will be automatically added to Publons, you can then choose whether or not to claim your Publons credit. I understand this statement.

Yes Choose an item.
