# Supplementary material for: Fluorescence microscopy datasets for training deep neural networks
Source: Gigascience. 2021 May 5;10(5):giab032. doi: 10.1093/gigascience/giab032 (PMC8099770; doi:10.1093/gigascience/giab032)
Supplement: giab032_Reviewer_2_Report_Original_Submission — Martin Weigert -- 7/23/2020 Reviewed [file giab032_reviewer_2_report_original_submission.pdf]

Reviewer Report

Title: Fluorescence Microscopy Datasets for Training Deep Neural Networks

Version: Original Submission **Date:** 7/23/2020

Reviewer name: Martin Weigert

Reviewer Comments to Author:

The paper by Hagen et al. provides fluorescence microscopy datasets to be used for training and evaluation of deep learning (DL) based denoising methods. Specifically, the authors gathered several image sets of corresponding low and high SNR 2D image pairs of endothelial (BPAE) cells with two different labelings (mitochondria and actin) and acquired with different microscopes (widefield and confocal). The paper further compares denoising results obtained with different DL based and classical methods.

Overall

Publicly available training datasets for DL methods are an important driver of research, yet compared to other fields (as computer vision) such datasets are currently less commonly found for fluorescence microscopy. So I really like that the paper tries to make a contribution towards changing that situation. I similarly like that the authors compared results from several DL methods as well as a strong classical baseline that are used in practice.

However the manuscript in its current form has two main issues:

1) The authors write that "High quality, publicly available data of this type has been lacking".

However there are some datasets that provide this (e.g. for 3D denoising [17]). Additionally, there is a recent publication [A] that provides such a dataset for seemingly the exact same situation (mixed poisson gaussian noise, 2D fluorescence microscopy images) yet with more diverse images (BPAE cells being a subset of it)

[A] Zhang et al. "A poisson-gaussian denoising dataset with real fluorescence microscopy images." CVPR. 2019.

So I wonder how much different the proposed dataset (and DL models trained on it) would be compared to [A]? Furthermore, for a public dataset to be valuable, the distribution of training images has to have a certain heterogeneity, such that evaluation on that data serves as a robust assessment of any method. The proposed dataset however contains only images of the same fixed sample (endothelial cells) of two essentially very stereotypical structures (Actin filaments and mitochondria). This makes it very hard to use the dataset for training models to be applied on differing structures (e.g. nuclei, membranes).

2) The current way of presenting the dataset/images (i.e. the main contribution) is suboptimal. Including at least an overview figure with a representative image for each modality/noise level/structure would greatly improve the paper (I essentially had to download the whole dataset just to have a look at a single image for each dataset). Additionally, Figure 1 has severe visual glitches that make it impossible to inspect the different denoising results. Finally, providing insets in the same figure for the denoised images would greatly help to see the differences of the compared methods.

Minor Comments:

- "Each dataset consisted of images of size 2048Ã—2048 pixels" -> Apart from dataset 4?
- The MSE formula on line 102 misses the lower limit in the sum ("j=0"?)
- "Following the standard implementation of the CSBDeep network, we used the Laplacian loss function" -> The default loss function in CSBDeep is mean absolute error MAE without any probabilistic component (the config default is probabilistic=False). The laplace loss should only be used if the resulting probabilistic model is needed (e.g. when the additional confidence prediction might be useful), which for a normal denoising task is not the case. I therefore would suggest to rerun at least some of the experiments with the default setting (probabilistic=False) and see whether the results change.
- BlindSpot: "uses careful padding and cropping to force the network to..." -> Padding and cropping is not really the main distinction of Blindspot networks.
- The relatively poor performance of the BlindSpot Network seems to me a bit surprising. "We used our own implementation in Python using the Keras library" ->
I think it would be more convincing when using one of the official implementations, e.g. https://github.com/juglab/n2v
- How was the parameter of BM3D (noise level sigma) tuned?
- "We normalized both images by clipping values below the 1st percentile and above the 99th percentile". Doesn't this remove essential information of the image? What was the reason to clip?
- What stopping criterion was used for the CARE/Blindspot network training?

Level of Interest

Please indicate how interesting you found the manuscript: Choose an item.

Quality of Written English

Please indicate the quality of language in the manuscript: Choose an item.

Declaration of Competing Interests

Please complete a declaration of competing interests, considering the following questions:

- Have you in the past five years received reimbursements, fees, funding, or salary from an organisation that may in any way gain or lose financially from the publication of this manuscript, either now or in the future?
- Do you hold any stocks or shares in an organisation that may in any way gain or lose financially from the publication of this manuscript, either now or in the future?
- Do you hold or are you currently applying for any patents relating to the content of the manuscript?

- Have you received reimbursements, fees, funding, or salary from an organization that holds or has applied for patents relating to the content of the manuscript?
- Do you have any other financial competing interests?
- Do you have any non-financial competing interests in relation to this paper?

If you can answer no to all of the above, write 'I declare that I have no competing interests' below. If your reply is yes to any, please give details below.

I declare that I have no competing interests

I agree to the open peer review policy of the journal. I understand that my name will be included on my report to the authors and, if the manuscript is accepted for publication, my named report including any attachments I upload will be posted on the website along with the authors' responses. I agree for my report to be made available under an Open Access Creative Commons CC-BY license (http://creativecommons.org/licenses/by/4.0/). I understand that any comments which I do not wish to be included in my named report can be included as confidential comments to the editors, which will not be published.

Choose an item.

To further support our reviewers, we have joined with Publons, where you can gain additional credit to further highlight your hard work (see: https://publons.com/journal/530/gigascience). On publication of this paper, your review will be automatically added to Publons, you can then choose whether or not to claim your Publons credit. I understand this statement.

Yes Choose an item.
